# Importance of Low Pregnancy Associated Plasma Protein-A (PAPP-A) Levels During the First Trimester as a Predicting Factor for Adverse Pregnancy Outcomes: A Prospective Cohort Study of 2636 Pregnant Women

**DOI:** 10.7759/cureus.31256

**Published:** 2022-11-08

**Authors:** Maria Papamichail, Zacharias Fasoulakis, George Daskalakis, Marianna Theodora, Alexandros Rodolakis, Panagiotis Antsaklis

**Affiliations:** 1 First Department of Obstetrics and Gynecology, National and Kapodistrian University of Athens School of Medicine, Athens, GRC; 2 First Department of Obstetrics and Gynecology, National and Kapodistrian University of Athens, Athens, GRC; 3 Obstetrics and Gynecology, National and Kapodistrian University of Athens, Athens, GRC

**Keywords:** first trimester screening, perinatal outcome, pregnancy associated plasma protein alpha, low serum papp-a, adverse pregnancy outcomes

## Abstract

Objectives: The aim of this study is to investigate the predictive value of low levels of pregnancy associated plasma protein-A (PAPP-A) during the first trimester on adverse pregnancy outcomes, namely pregnancy induced hypertension (PIH), preeclampsia (PE), intrauterine growth restriction (IUGR), and fetal loss.

Methods: This is a prospective cohort study including 2636 women with singleton pregnancies that attended the Prenatal Diagnosis Unit of the First Department of Obstetrics and Gynecology of National and Kapodistrian University of Athens in “Alexandra Hospital” between 2017 and 2019 for the first trimester combined screening routine scan. The study population was divided into two groups according to their PAPP-A levels. The cut-off value of the PAPP-A level was defined as the 0.4 multiple of median (MoM) which is in correspondence with the fifth centile. The women were followed-up prospectively until delivery and the primary outcome measures were the incidence of PIH, PE, IUGR (<10th centile), and fetal loss.

Results: PAPP-A levels of ≤0.4 MoM were associated with increased maternal body mass index (BMI), increased uterine arteries pulsatility index (PI), and lower birth weight. Women with PAPP-A levels ≤0.4 MoM were more likely to develop PE (2.3% vs. 0.2%, p<0.001), PE or PIH (2.3% vs. 0.4%, p=0.003), IUGR (2.3% vs. 0.4%, p=0.003), and combined adverse outcome (25.5% vs. 1.9%, p<0.001) compared to women with PAPP-A>0.4 MoM. Conclusions: This study confirms that among women with PAPP-A levels ≤0.4 MoM in the first trimester, there are increased odds for PE or PIH, IUGR, and combined composite pregnancy outcome.

## Introduction

Pregnancy-associated plasma protein-A (PAPP-A) is a protease that belongs to the family of matrix metalloproteinases and its encoding gene is located on chromosome 9q33.1 [1]. Although it has been identified in many human tissues such as the colon, kidneys, breast, and bone marrow, a greater proportion of PAPP-A is present in the serum of pregnant women, as it is produced by the syncytiotrophoblast of the placenta in an increased quantity. PAPP-A’s role is to release the insulin-like growth factor (IGF) from its binding protein, and this effect is greater at IGFBP-4. Hence, IGF is free to express its actions to mitogenic activity, differentiation, and placental invasion. Moreover, as IGF’s bioavailability increases by PAPP-A, a significant local proliferative cellular response is induced, and transportation of glucose and amino acids in the placenta is achieved, playing a crucial role in normal fetal development and pregnancy progress [2-3]. According to Fialova and Malbohan [4], PAPP-A starts to be detectable during early pregnancy soon after blastocyst implantation and its levels continue an upward trend throughout pregnancy until delivery and its maximum concentration is obtained at term.

Pregnancy-associated plasma protein-A was initially introduced by Lin et al. in 1974 [5] and since then it has been established as one of the markers in the combined screening test of the first trimester for the screening of chromosomal aneuploidies. Nevertheless, many pregnancies with decreased PAPP-A levels have a negative test for chromosomal abnormalities. This occurs mainly in younger women (<30 years) with low nuchal translucency (NT<1.5 mm) [6]. Therefore, many studies in the current literature provided data indicating that as PAPP-A is a placental product, low maternal serum concentration of PAPP-A in the first trimester of pregnancy is correlated to placental dysfunction and thus it is an important predictor for the outcome of pregnancy [7].

In this study, the predictive value of first trimester PAPP-A levels regarding the risk for preeclampsia (PE), pregnancy induced hypertension (PIH), intrauterine growth restriction (IUGR), cesarean section, and fetal and neonatal death, are investigated. This information would be extremely helpful to enable accurate and timely counseling of women at risk for adverse pregnancy outcomes, providing them with the best antenatal care.

## Materials and methods

This is a prospective cohort study including 2636 women that attended the Prenatal Diagnosis Unit of the First Department of Obstetrics and Gynecology of National and Kapodistrian University of Athens in “Alexandra Hospital” between 2017 and 2019 for the first-trimester routine screening test for aneuploidies. The inclusion criteria were both nulliparous and multiparous women with singleton pregnancies, gestational age between 11 and 14 weeks, and crown-rump length (CRL) measuring from 45 to 84 mm. Gestational age was calculated from the first day of the last menstrual period (LMP) and confirmed by CRL measurement. The vast majority of the participants were Greek. Exclusion criteria were: multiple pregnancies, chromosomal abnormalities, major congenital anomalies, or fetal loss diagnosed at the time of the examination. Additionally, women lacking any data included in the statistical analysis were also excluded from the study. Blood sampling was performed on the same day as the ultrasound scan. Serum maternal PAPP-A was measured in the department’s laboratory as part of the first trimester screening and was adjusted for gestational age, maternal weight, parity, ethnicity, smoking, maternal diabetes, and use of artificial reproductive technologies (ART). PAPP-A levels were measured with an automated immunofluorescent assay (B∙R∙A∙H∙M∙S KRYPTOR, ThermoScientific, Hennigsdorf, Germany) and were expressed in multiple of median (MoM). Doppler examination of the uterine arteries was performed using transabdominal ultrasound with color flow mapping. All participating obstetricians are certified by the Fetal Medicine Foundation for first-trimester screening and Doppler measurements. The participants were divided into two groups according to their PAPP-A levels at the time of the first-trimester scan. The cut-off value of the PAPP-A level was defined as 0.4 MoM which corresponds with the fifth centile. This measurement was selected because this cut-off point was found to be the most representative of our study’s population. The first group included women with PAPP-A levels ≤ 0.4 MoM (cases) and the control group women with PAPP-A < 0.4 MoM.

Women were followed-up prospectively until delivery and the primary outcome measures were the incidence of PIH, PE, IUGR, fetal loss, and cesarean section. Outcomes were extracted either by phone calls to the mothers or from the clinic records by a physician blinded to PAPP-A results. PE was defined according to the International Society of Hypertension in Pregnancy criteria: the presence of hypertension in pregnancy or two readings of systolic arterial pressure (SAP) ≥ 140 mmHg and/or diastolic arterial pressure (DAP) ≥ 90 mmHg in a 6 h interval with proteinuria of ≥300 mg in 24 h or 2+ on urine dipstick analysis. PIH was defined as the occurrence of two readings of SAP ≥ 140 mmHg and/or DAP ≥ 90 mmHg in a 6 h interval in a pregnant woman without pre-existing hypertension and proteinuria. IUGR was defined as the estimated fetal weight (EFW) below the 10th centile.

Statistical analysis

Statistical significance was set at 0.05 and analyses were conducted using SPSS statistical software (version 22.0) (IBM Inc., Armonk, NY). Continuous variables are expressed as mean and standard deviation (SD). Qualitative variables were expressed as absolute and relative frequencies. The normal distribution was evaluated using the Kolmogorov-Smirnov test. For the comparisons of proportions, chi-square and Fisher’s exact tests were used. Student’s t-tests were computed for the comparison of mean values between the two study groups. Sensitivity, specificity, and negative and positive predictive values were determined for evaluating the predictive ability of MoM PAPP-A for adverse outcomes. Additionally, logistic regression analysis was used to calculate odds ratios (ORs) and 95% confidence intervals (95% CIs) for the prediction of adverse outcomes. All p-values reported are two-tailed.

Ethical approval

The study was approved by the institutional board of the Medical School of National and Kapodistrian University of Athens in association with the First Department of Obstetrics and Gynecology numbered 423/12-12-2016. All participants were also informed for the scope of the study.

## Results

The study included 2636 women. The sample’s mean age was 34.3 years (SD=4.7), and the mean maternal body mass index (BMI) was 24.3 (SD=7.5). One hundred fifty-five women had a personal medical history, with most of these women (65%) having a previous cesarean section while 41.3% had other prior uterine surgeries. Sample characteristics are presented in Table 1.

**Table 1:** Sample characteristics. BMI, body mass index; SD, standard deviation; CRL, crown-rump length; PI, pulsatility index; MoM PAPP-A, multiple of median pregnancy-associated plasma protein-A; IUGR, intrauterine growth restriction; PE, preeclampsia

Sample characteristics	N (%)
Maternal BMI (kg/m2), mean (SD)	24.3(7.5)
CRL, mean (SD)	65.2(8)
PI left, mean (SD)	1.54(0.54)
PI right, mean (SD)	1.56(0.89)
Infant sex	
Boys	1388(53.1)
Girls	1228(46.9)
Birth weight (g), mean (SD)	3166(518)
MOM PAPP-A	
≤0.4	256(9.7)
>0.4	2380(90.3)
Delivery	
Vaginal delivery	1366(52.7)
Cesarean section	1174(45.3)
Vacuum-assisted delivery	50(1.9)
Outcome	
Born alive with adverse event	2576(97.7)
Fetal loss	26(1)
Neonatal death	2(0.1)
PE	10(0.4)
Hypertension	6(0.2)
IUGR	16(0.6)

The mean birth weight was 3166 g (SD=518). Fetal loss and neonatal death were recorded in 1% and 0.1% of the sample, respectively. Six women (0.2%) had hypertension and 0.4% had PE. IUGR fetuses were recorded in 0.6% of the sample.

Pregnancy associated plasma protein-A less than 0.4 MoM was found in 256 cases (9.7%) and the rest of the sample (90.3%) had PAPP-A more than 0.4 MoM and consisted of the control group. Maternal BMI and both left and right pulsatility index (PI) of uterine arteries were significantly higher in the group with PAPP-A less than 0.4 MoM. Also in the group with low PAPP-A was recorded significantly lower birth weight (3000 g vs. 3183.5 g, p<0.001). Table 2 shows the characteristics of the cases and controls.

**Table 2:** Characteristics of the two study groups. BMI, body mass index; CRL, crown-rump length; PI, pulsatility index; MoM PAPP-A, multiple of median pregnancy associated plasma protein-A; SD, standard deviation +Student’s t-test; ++Fisher’s exact test

	Group		p
	Cases MoM PAPP-A ≤ 0.4	Controls MoM PAPP-A > 0.4	
	Mean (SD)	Mean (SD)	
Maternal BMI (kg/m2)	25.5 (8.7)	24.2 (7.3)	0.006+
CRL	65.3 (7.4)	65.2 (8.1)	0.908+
PI left	1.6 (0.6)	1.5 (0.5)	0.041+
PI right	1.7 (0.6)	1.5 (0.9)	0.019+
Birth weight	3000 (584.7)	3183.5 (506.9)	<0.001+
Delivery, N (%)			
Vaginal delivery	98 (39.2)	1268 (54,2)	<0.001++
Cesarean section	148 (59.2)	1026 (43.8)	
Vacuum-assisted delivery	4 (1.6)	46 (2)	

**Figure 1:**
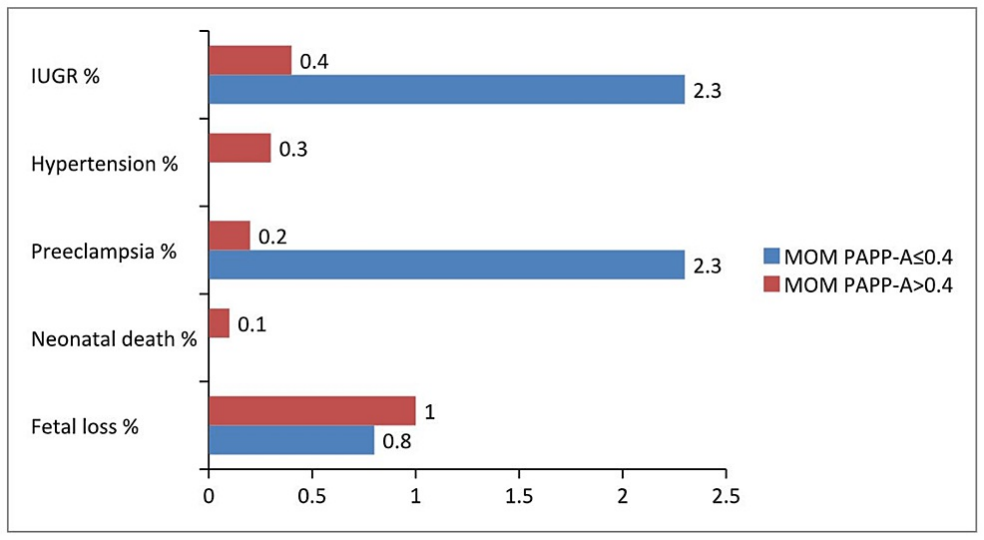
Adverse outcomes in cases and controls. IUGR, intrauterine growth restriction; MoM, multiple of the median; PAPP-A, pregnancy associated plasma protein-A

Comparing all adverse outcomes between cases and controls (Table 3), it was found that women with low PAPP-A levels had a greater proportion of PE (2.3% vs. 0.2%, p<0.001) and IUGR (2.3% vs. 0.4%, o=0.003) (Figure 1). Also, the combined outcome of PE or hypertension was more frequent in cases rather than controls (2.3% vs. 0.4%, p=0.003). As far as it concerns the composite outcome of fetal loss, neonatal death, PE, hypertension and IUGR, it was found a significantly greater proportion in cases than in controls (5.5% vs. 1.9%, p<0.001). Interestingly, in the group with PAPP-A levels below 0.4 MoM no incidence of PIH was recorded, while in the control group, 0.3% of the participants (6/2380) developed PIH. PIH, fetal loss, and neonatal death were not found to be statistically significant when they were analyzed individually. ORs for the prediction of PE, IUGR, and PE or hypertension from low MoM PAPP-A (Table 4) were significant and equal to 14.26, 5.69 and 5.69, respectively. Women with low MoM PAPP-A had 2.94 times greater odds of having a composite outcome (fetal loss, neonatal death, PE, hypertension, and IUGR). Sensitivities were 23.3% for the composite outcome, 37.5% for both IUGR and PE/PIH, and 60% for PE. Specificities were higher and ranged from 90.5% to 90.6% for PE, PE/PIH, IUGR, and composite outcome respectively (Τable 4).

**Table 3:** Comparison of all adverse outcomes between cases and controls. PE, preeclampsia; IUGR, intrauterine growth restriction Note: Composite outcome refers to fetal loss, neonatal death, PE, hypertension, and IUGR ^+^Pearson’s Chi square test; ^++^Fisher’s exact test

	Group				
	Cases MOM PAPP-A ≤ 0.4		Control MOM PAPP-A > 0.4		
	N	%	N	%	P
Outcome					
Born alive without adverse outcome	242	94.5	2334	98.1	<0.001^+^
Fetal loss	2	0.8	24	1.0	1.000^++^
Neonatal death	0	0.0	2	0.1	1.000^++^
PE	6	2.3	4	0.2	<0.001^++^
Hypertension	0	0.0	6	0.3	1.000^++^
PE/hypertension	6	2.3	10	0.4	0.003^++^
IUGR	6	2.3	10	0.4	0.003^++^
Composite outcome	14	5.5	46	1.9	<0.001^+^

 

**Table 4:** Diagnostic indices and ORs for the prediction of outcomes from MoM PAPP-A ≤ 0.4. Note: Composite outcome refers to fetal loss, neonatal death, PE, hypertension, and IUGR PPV, positive predictive value; NPV, negative predictive value; ORs, odds ratios; CI, confidence interval; PE, preeclampsia; IUGR, intrauterine growth restriction

	Sensitivity (%)	Specificity (%)	PPV (%)	NPV (%)	OR (95% CI)	p
PE	60.0	90.5	2.3	99.8	14.26 (3.99–50.85)	<0.001
IUGR	37.5	90.5	2.3	99.6	5.69 (2.05–15.78)	0.001
PE/hypertension	37.5	90.5	2.3	99.6	5.69 (2.05–15.78)	0.001
Composite outcome	23.3	90.6	5.5	98.1	2.94 (1.59–5.42)	0.001

## Discussion

The data in this current study show that PAPP-A levels below 0.4 MoM in the first trimester -- as a single maternal serum marker -- are able to identify pregnancies at an increased risk for PE, IUGR, PIH, and occurrence of any adverse pregnancy outcome (PE, PIH, IUGR, fetal and neonatal death) compared to the control population. Most importantly, the correlation between low PAPP-A levels and PE, PIH, IUGR, and the composite adverse outcome was strong. These findings are in agreement with many previous studies in the current literature [7-11]. The biggest trial has been run by Dugoff et al. [8], the FASTER trial, which studied 34,271 pregnancies and explored the predictive value of PAPP-A, β-hCG, and NT on adverse pregnancy outcomes. The authors concluded that PAPP-A levels below 0.4 MoM or ≤ fifth percentile, when analyzed as a single marker, are correlated with spontaneous fetal loss, low birth weight, PE, gestational hypertension, preterm birth, preterm premature rupture of membranes, and placental abruption. Morris et al. [9] in their meta-analysis including 175,240 pregnancies, calculated that PAPP-A levels below the fifth centile could predict neonates with birth weight <10th centile, neonates with birth weight <5th centile, PE, preterm birth <37 weeks of gestation, and composite adverse outcome. However, the predictive ability of low PAPP-A levels in that study was moderate. D’Antonio et al., also designed an important study exploring the incidence of adverse pregnancy outcomes among women with low PAPP-A levels in the first trimester, concluding that PAPP-A levels in mothers experiencing pregnancy complications and more specifically PE, early PE, IUGR, and preterm birth are statistically significantly lower compared to mothers enjoying an uncomplicated gestation. Moreover, neonates whose mothers had low PAPP-A levels in the first trimester, are more likely to present lower Apgar scores and a higher possibility for NICU admission [7, 10-15]. Table 5 summarizes some of the most important studies exploring the predicting value of low PAPP-A levels in the first trimester for adverse pregnancy outcomes.

**Table 5:** Summary of studies and their outcomes. CRL, crown-rump length; PE, preeclampsia; IUGR, intrauterine growth restriction

Authors, year, study type	Number of participants, eligible women	Statistical significant outcomes	Non-statistical significant outcomes	Conclusions, authors’ suggestions
Dugoff et al. (2004) [8], Prospective cohort study	n=33 395 Inclusion criteria: viable singleton pregnancy with fetal CRL between 36 and 79 mm Exclusion criteria: anencephaly, cystic hygroma, chromosomal or structured abnormality, insulin – dependent diabetes mellitus	PAPP-A ≤5^th^ centile Spontaneous loss at ≤24 weeks of gestation intrauterine fetal demise at >24 weeks of gestation preterm birth at <37 or ≤ 32 weeks of gestation PIH PE birth weight <10^th^ or ≤5^th^ centile premature rupture of membranes placental abruption	PAPP-A ≤5^th^ centile neonatal death placenta previa gestational diabetes mellitus	Although low PAPP-A levels are associated with adverse pregnancy outcomes, sensitivity and positive predictive value are low. Thus, PAPP-A as a single marker is a poor predictor for adverse pregnancy outcomes.
Morris et al. (2017) [9], Meta-analysis	n= 175 240 Heterogenity of inclusion and exclusion criteria	PAPP-A <5^th^ centile birth weight <10^th^ or ≤ 5^th^ centile preterm birth pregnancy loss < 24 weeks PE composite adverse outcomes	PAPP-A <5^th^ centile stillbirth >24 weeks of gestation	PAPP-A levels <5^th^ centile in the first trimester are associated with adverse pregnancy outcomes, but predictive values are poor. There is no evidence suggesting any intervention in women with low PAPP-A levels, as the vast majority of these women will experience an uncomplicated pregnancy
D’Antonio et al. (2013) [7], retrospective observational study	n= 12 355 inclusion criteria: viable singleton pregnancies exclusion criteria: multiple pregnancies, non-viable pregnancies	PAPP-A <5^th^ centile IUGR preterm birth PE and early PE composite adverse outcomes		PAPP-A levels in mothers developing PE, IUGR, and who delivering preterm, are lower compared to mothers who experience an uncomplicated pregnancy. Nevertheless, PAPP-A as a single marker is a poor predictor for these conditions.
Bilagi et al. (2017) [16] retrospective cohort study	n=12 837 inclusion criteria: viable singleton pregnancies with CRL 45-84 mm	Lower PAPP-A levels are associated with IUGR preterm birth PE	Lower PAPP-A levels are not associated with stillbirth miscarriage perinatal death neonatal death	Further work is needed for the assessment of the predicting ability of PAPP-A levels in adverse pregnancy outcomes.

Moreover, many studies [7-9, 15-19] calculated also the predictive value of PAPP-A levels ≤1st centile, resulting in a stronger correlation with any adverse pregnancy outcomes. Kaijomaa et al. [12] run a cohort in order to investigate the predictive value of extremely low PAPP-A levels in the first trimester (below 0.3 MoM) for adverse pregnancy outcomes. The authors concluded that the risks for aneuploidies, structure abnormalities, preterm birth, and IUGR in women with PAPP-A levels <0.1 MoM were 6-, 36-, 1.8-, and 3.8 fold increased respectively compared to women with PAPP-A levels of 0.2-0.3 MoM. Therefore, the lower the PAPP-A MoM, the higher are the odds for developing adverse pregnancy outcomes.

One of the most important results of this study is that the correlation between low PAPP-A levels and PE, PE or PIH, IUGR, and composite adverse outcome is strong (OR 14.26, 5.69, 5.69, and 2.94 respectively). This finding contradicts most of the studies [7, 9, 13-14] which agree that low PAPP-A levels are a poor predictor for adverse pregnancy outcomes and, therefore, should not be used as a single marker for these conditions. Poon et al., in their trial, calculated the predictive value of PAPP-A levels and mean PI of uterine arteries in the first trimester for PE prediction and found this correlation statistically significant. However, the predictive value was not different without the contribution of PAPP-A [15]. According to Morris et al., “the vast majority of women with low PAPP-A levels will have a normal pregnancy outcome and the majority of women with an adverse outcome will have a normal PAPP-A” [9]. Despite the fact that in our study the correlation between low PAPP-A and any adverse pregnancy outcomes is stronger, further studies are needed to confirm the necessity of any intervention in this women’s population.

Strengths and limitations

This study meets several strengths. The most important of them are the prospective design of the study and the large number of participants who were routinely screened women, making the study’s sample representative of the general population. Also, pregnancies complicated with chromosomal or structured anomalies were excluded and the outcomes were collected by a blinded physician, making the predictive values of the test more accurate and unbiased. Nevertheless, it would be an omission not to mention the limitations of the study. In the design of the study were not included the medical and obstetrical history of the mothers and their smoking status. Concerning the outcomes, preterm birth and premature rupture of the membranes were not included in the analysis. In addition, some of the outcomes were collected via a phone call questionnaire and therefore this information was prone to bias.

## Conclusions

This study confirms that among women with PAPP-A levels ≤0.4 MoM in the first trimester, there are increased odds for PE or PIH, IUGR, and combined composite pregnancy outcome. PE represents one of the most feared pregnancy complications, carrying a heavy psychological and socioeconomic impact on the family. Despite the fact that there is no consensus regarding the predicting ability of low PAPP-A levels in the first trimester for adverse pregnancy outcomes and the benefit of any intervention in this women’s population, closer fetal surveillance and mother antenatal monitoring would be beneficial.
